# Dr. Robert Jones and the Mentally Defective

**Published:** 1912-04-13

**Authors:** 


### DR. ROBERT JONES AND THE MENTALLY DEFECTIVE.

It is fortunate that some subjects which deeply concern the true welfare of the nation do not appeal to the minds of men who are mere politicians. It is also to be deplored that for the same reason measures for which the country is ripe and upon the principles of which moderate men of all shades of opinion are agreed, should be so long delayed. Foremost among such questions is that of the treatment of the mentally defective. Dr. Robert Jones, the medical superintendent of the Claybury Mental Hospital, in his recent letter to the *Times* is but voicing the settled opinions of thousands of men and women who are spending themselves, their time, and their money in dealing with those who come under that category.

The Act of 1899 provides for the education of defective and epileptic children from the age of five to sixteen. It has proved beneficial. The ordinary schools are better without the defectives. The defectives themselves receive special attention and learn something. What is perhaps the best feature of the system is that they can be kept under notice by those interested in them and assisted after leaving school. It is, however, felt that then is the time when it is so important that they should be looked after and safeguarded. For this there is at present no provision. Dr. Jones suggests that the Act, which is optional, should be made compulsory in all urban areas which contain above a certain population, and that after leaving school the children should be sent to homes or colonies which are to be "Hives of Industry." The necessity for such places of refuge is generally acknowledged. We are not, however, altogether in agreement with the suggestion that these colonies or homes should be in direct proximity to the county or borough mental hospital of the district. We fully appreciate the point that the head of such an institution should be imbued with and actuated by that spirit which has made our mental hospitals places where the insane receive "the highest scientific as well as the most humane treatment in any country." Such homes must, however, not run any risk of becoming merely an additional block in the extended mental-hospital grounds. They must be as free as possible from the asylum atmosphere. They must be schools rather than hospitals for mental disease.

Each authority upon whom the obligation rests to provide accommodation for lunatics should be compelled to provide separate wards for idiot children who are either ineducable or are unfit to associate with those who could be taught. Such children in time would become inmates of the adult wards. Neighbouring authorities should unite to provide an institution for those capable of improvement. It could then be of such a size as would justify expenditure upon its surroundings and means of education. We are no advocates for spending public money in vain efforts to reach the unattainable, and there are many of the mentally defective with regard to whom it is worse than useless to attempt much. Under any system of segregation and safeguarding the advantage will be not so much to the individual defective, though he too will distinctly benefit, as to the community. How pressing is the need for action, and at the same time how potent in the face of such necessity is the reluctance to certify, is shown by the following quotation from the Report of the Commissioners in Lunacy: "In one county workhouse the visiting Commissioner found living in the house, uncertified and therefore free to go in and out as they please, three women of this class who had between them had eleven illegitimate children, and three others awaiting confinement, one of whom had already had five illegitimate children. We cannot help thinking that the reluctance to certify cases of this class arises largely from inexperience of the local medical officers as to the requirements of certifiability, and also from what we consider mistaken notions of kindness in the wish to avoid the stigma of insanity." Some of the cases which give most thought to reformers and most trouble to administrators may escape the net. The recommendation of the Royal Commission with regard to the mentally defective who become criminal is most important. The power to examine their mental state and deal with them at the beginning of their criminal career is most necessary and most humane.

Yet in all our efforts to deal with this question let us not forget one fundamental fact. The Royal Commission arrived at the conclusion that feeble-mindedness is largely inherited. By detaining defectives, and especially defectives of the higher types, in institutions under supervision much good may be done. How much even then would still remain to be done is known best to those who work among the insane. We have before us a family tree which is one among many, and can be paralleled from the records of any asylum, that shows what is going on. When we are educated sufficiently to distinguish clearly between what is good for the race and what is mistaken and sentimental kindness to the individual, we shall go further than even the most ardent reformer would go to-day.

